# Oral Fluid Concentrations of Tenofovir and Emtricitabine for Monitoring HIV Antiretroviral Adherence: Findings From a Randomized Trial with Directly Observed Therapy (TARGET Study)

**DOI:** 10.1093/ofid/ofaf766

**Published:** 2025-12-12

**Authors:** Xin Niu, Tiancheng E Edwards, Jeannette A Dienhart, Richard E Haaland, Oraphan Siriprakaisil, Pra-ornsuda Sukrakanchana, Tim R Cressey, Paul K Drain

**Affiliations:** Department of Epidemiology, University of Washington, Seattle, Washington, USA; Department of Global Health, International Clinical Research Center, University of Washington, Seattle, Washington, USA; Division of HIV Prevention, Centers for Disease Control and Prevention, Atlanta, Georgia, USA; Division of HIV Prevention, Centers for Disease Control and Prevention, Atlanta, Georgia, USA; Division of HIV Prevention, Centers for Disease Control and Prevention, Atlanta, Georgia, USA; Sanpatong Hospital, Chiang Mai, Thailand; AMS-PHPT Research Collaboration, Faculty of Associated Medical Sciences, Chiang Mai University, Chiang Mai, Thailand; AMS-PHPT Research Collaboration, Faculty of Associated Medical Sciences, Chiang Mai University, Chiang Mai, Thailand; Department of Pharmacology and Therapeutics, University of Liverpool, Liverpool, UK; Department of Epidemiology, University of Washington, Seattle, Washington, USA; Department of Global Health, International Clinical Research Center, University of Washington, Seattle, Washington, USA; School of Medicine, University of Washington, Seattle, Washington, USA

**Keywords:** adherence, emtricitabine, oral fluids, pre-exposure prophylaxis, tenofovir

## Abstract

**Background:**

Early identification of non-adherence to daily antiretroviral therapies may facilitate targeted transition to long-acting HIV regimens for individuals most likely to benefit. This study evaluated the feasibility of detecting emtricitabine (FTC) and tenofovir (TFV) in oral fluids to monitor daily oral pre-exposure prophylaxis (PrEP) adherence.

**Methods:**

We collected 241 oral fluids from 22 HIV-negative adults randomized to 2, 4, or 7 doses/week of oral PrEP with FTC/TDF for directly observed therapy. Generalized Estimating Equations were used to examine the associations between FTC/TFV detectability and three dimensions of PrEP adherence: dosing recency, cumulative dosing time, and dosing frequency. We also assessed the diagnostic accuracy of FTC levels in oral fluids for predicting daily oral PrEP non-adherence (time since the last dose >24 hours).

**Results:**

Among 165 oral fluids with a time since the last dose within 48 hours, 9.0% had detectable TFV, and 53.3% had detectable FTC. Compared with oral fluids collected within 24 hours since the last dose, those collected between 24 and 48 hours since the last dose had significantly lower odds of having detectable FTC (odds ratio: 0.21, 95% confidence interval [CI]: 0.11–0.38). An FTC threshold of <7.5 ng/mL achieved a sensitivity of 90% (95% CI: 84%–94%) and a specificity of 65% (95% CI: 57%–74%) for identifying recent PrEP non-adherence.

**Conclusions:**

FTC can be detected in oral fluids and may be a promising pharmacologic marker for identifying recent PrEP non-adherence as early as one dose missed. TFV had lower concentrations in oral fluids, and is not suitable for adherence monitoring.

**Clinical Trials Registration.** NCT0301260.

## Teaser

Emtricitabine detection in oral fluids is highly sensitive to identify recent non-adherence (monitor adherence) to daily oral PrEP and may help guide timely, targeted interventions for individuals at risk of poor adherence.

Daily oral pre-exposure prophylaxis (PrEP) demonstrated high efficacy against HIV-1 acquisition, and there are more than 2.6 million users worldwide [1]. The United Nations' political declaration on Ending AIDS by 2030 established an ambitious yet achievable target for national governments to ensure PrEP access for 10 million people by 2025 [2]. The fight against HIV has seen significant progress with the growing availability and diversification of PrEP medications/regimens such as long-acting cabotegravir (CAB-LA), lenacapavir (LEN) or event-driven (ED) PrEP. These advancements now offer a wider range of effective options, catering to different individual needs/preferences of people at risk for HIV infections. While daily oral PrEP with emtricitabine/tenofovir disoproxil fumarate (FTC/TDF) remains one of the first-line options recommended by the World Health Organization (WHO) [3], its effectiveness is highly dependent on consistent and timely medication adherence [4, 5]. Unfortunately, non-adherence continues to be a global challenge with studies showing high discontinuation rates (41% within six months) [6] and suboptimal adherence (only 29% maintaining ideal daily dosing) [4].

PrEP adherence monitoring can help identify individuals who struggle with daily oral regimens early on, providing valuable information for implementing targeted support interventions to improve adherence. Detecting non-adherence may also highlight the need to explore alternative PrEP options, such as CAB-LA or LEN, which may better meet users' needs. Compared with traditional methods for adherence monitoring such as self-reporting, pill counts or pharmacy refills, direct pharmacologic measurement of biological samples provides a more accurate reflection of real-time drug concentrations and is less susceptible to social desirability or recall bias [7–9]. Pharmacologic metrics of adherence not only can guide interventions, but also predict key HIV prevention and treatment clinical outcomes, including future seroconversion events and virologic suppression, and has become critically important for understanding variations observed in trial efficacy [10–13].

Pharmacologic metrics of PrEP drug metabolites provide different insights into adherence based on their half-lives in various biological samples. Tenofovir-diphosphate (TFV-DP) in dried blood spots (DBS) has a long half-life and is effective for assessing cumulative adherence (dosing frequency after the steady state is achieved) over the preceding 6–12 weeks [14]. In contrast, plasma and urine tenofovir (TFV) do not accumulate significantly due to their short half-life but provide valuable information about when the last PrEP dose was taken (dosing recency) [15, 16]. This can help understand adherence patterns before achieving steady state or when steady state is not maintained due to inconsistent dosing intervals from non-adherence.

Compared with blood and urine, oral fluid is less invasive, easier-to-collect and requires minimal privacy. These characteristics make oral fluid a desirable biological sample, especially for high-risk populations where rapid HIV self-tests based on oral fluids are already highly accepted and recommended as an innovative alternative strategy to increase access to and effective use of PrEP by the WHO [17]. However, there is limited research on the detectability of FTC/TFV in oral fluids [18–20]. The relationship between PrEP adherence and drug concentrations in oral fluids also remains underexplored. To address these gaps, we tested oral fluids collected from a randomized trial of FTC/TDF with directly-observed therapy (DOT). Our objectives were to (1) evaluate the relationships between drug concentrations in oral fluids and three dimensions of PrEP adherence (dosing recency, cumulative dosing time, and dosing frequency), (2) establish benchmarks and assess the diagnostic performance of FTC/TFV in oral fluids for predicting PrEP non-adherence, and (3) estimate the correlations between FTC/TFV concentrations in time-matched oral fluids and plasma.

## METHODS

### Study Design

The TARGET study, an open-label randomized pharmacokinetic (PK) trial, prospectively recruited healthy adults aged 18–49 years in Thailand for controlled levels of PrEP adherence. Details of the study protocol have been described elsewhere [21]. In brief, to represent low, moderate, and perfect PrEP adherence respectively, eligible participants were randomized (1:1:1) to receive 2, 4, or 7 doses/week of TDF 300 mg/FTC 200 mg (Truvada, Gilead Sciences) for 6 weeks. We performed DOT to ensure medication adherence and recorded the time for each drug intake during the 6-week dosing phase. All participants were followed for another 4 weeks (the washout phase) after their last dose.

### Sample Collections

Three time-matched plasma and oral fluids were collected from participants before the next scheduled dose during the dosing phase (on the Mondays of week 1/3/5). During the washout phase, seven time-matched collections were performed during week 7. Of note, one additional collection of oral fluids was scheduled 24 hours after the last dose at the start of week 7. Oral fluids (1 mL) were collected using the ACCU.SAL^TM^ (*Oasis Diagnostics*) devices. Plasma samples were collected from a single venous blood draw (5 mL) after centrifugation.

### Measurement of Drug Concentrations

Oral fluids and plasma samples were stored at −70°C until testing for PrEP drug levels. Drug concentrations were measured using validated high performance liquid chromatography tandem mass spectrometry (LC-MS/MS) based on previously published methodology [22]. The limit of quantification (LOQ) for both TFV and FTC was 10 ng/mL in oral fluids and plasma. The limit of detection (LOD) was 10 ng/mL for both drug molecules in plasma, and 5 ng/mL in oral fluids. To minimize bias, all laboratory testing (except for the pilot testing) was conducted in a blinded fashion regarding the dosing time of each sample. Consistent with prior research [20, 22], TFV and FTC was undetectable (below corresponding LODs) in all oral fluids collected more than 48 hours post-dose in our pilot testing of 20 randomly selected samples. Consequently, only the subset of matched samples with a time since the last dose within 48 hours was selected for testing.

### Statistical Analyses

Stratified by randomization arms, we calculated descriptive statistics [mean, median, interquartile range (IQR), standard deviation (SD)] for the baseline participant characteristics. Dosing recency (hours since the last dose taken), cumulative dosing time (weeks since the first dose), and dosing frequency (number of doses per week or randomization arm) were tabulated as three dimensions to describe PrEP adherence. Drug concentrations below corresponding LOD were considered as not detected. All readouts below LOQ but above LOD with discernable peaks were imputed as half of the corresponding LOQ for statistical calculations.

To account for repeated measures from the same participant, we performed binomial Generalized Estimating Equations (GEE) to assess the relationships between FTC and TFV detectability (drug levels detected > LOD) in oral fluids and the three dimensions of PrEP adherence. Dosing recency, cumulative dosing time and dosing frequency were respectively modeled as a binary variable (having a time since the last dose ≤24 hours vs between 24 and 48 hours), a continuous variable indicating weeks of dosing (1–6 weeks), and a categorical variable (2 vs 4 vs 7 doses/week). We plotted bar-plots and scatterplots of oral fluid drug concentrations by dosing recency.

To assess the diagnostic accuracy of FTC/TFV in oral fluids for predicting PrEP non-adherence, we calculated sensitivities and specificities for recent non-adherence using ROC (Receiver Operating Characteristic) analysis of clustered data. Since only samples collected within 48 hours since the last dose were selected for testing, recent non-adherence was defined as either “having a time since the last dose of more than 24 hours (up to 48 hours)” or was considered “missing the last dose of daily oral PrEP”. This definition served as the positive condition for prediction in the ROC analysis. We computed the 95% confidence intervals (CI) with stratified bootstrap. To establish a more real-world definition of recent non-adherence that accounts for more than one missed dose, we included all untested oral fluids collected more than 48 hours post-dose in our secondary ROC analysis. We assumed TFV/FTC would be undetectable in these samples based on results from our pilot testing and previously published studies.

## RESULTS

### Participant/Specimen Characteristics

We collected 241 oral fluids from 22 participants (41% female) (Supplementary Table 1). Since none of the oral fluid specimens with a time since the last dose >48 hours had detectable TFV or FTC in our pilot testing of 20 unblinded samples, a final subset of 165 (68.5%) oral fluids with a time since last dose ≤48 hours were tested for TFV/FTC. Among those tested, 143 (86.7%) had time-matched plasma samples collected. Fifty (30.3%) of the 165 oral fluids had a time since the last dose >24 hours while 115 (69.6%) collected within 24 hours post-dose (Supplementary Table 2).

### Drug Concentrations in Oral Fluids

**Figure 1.**
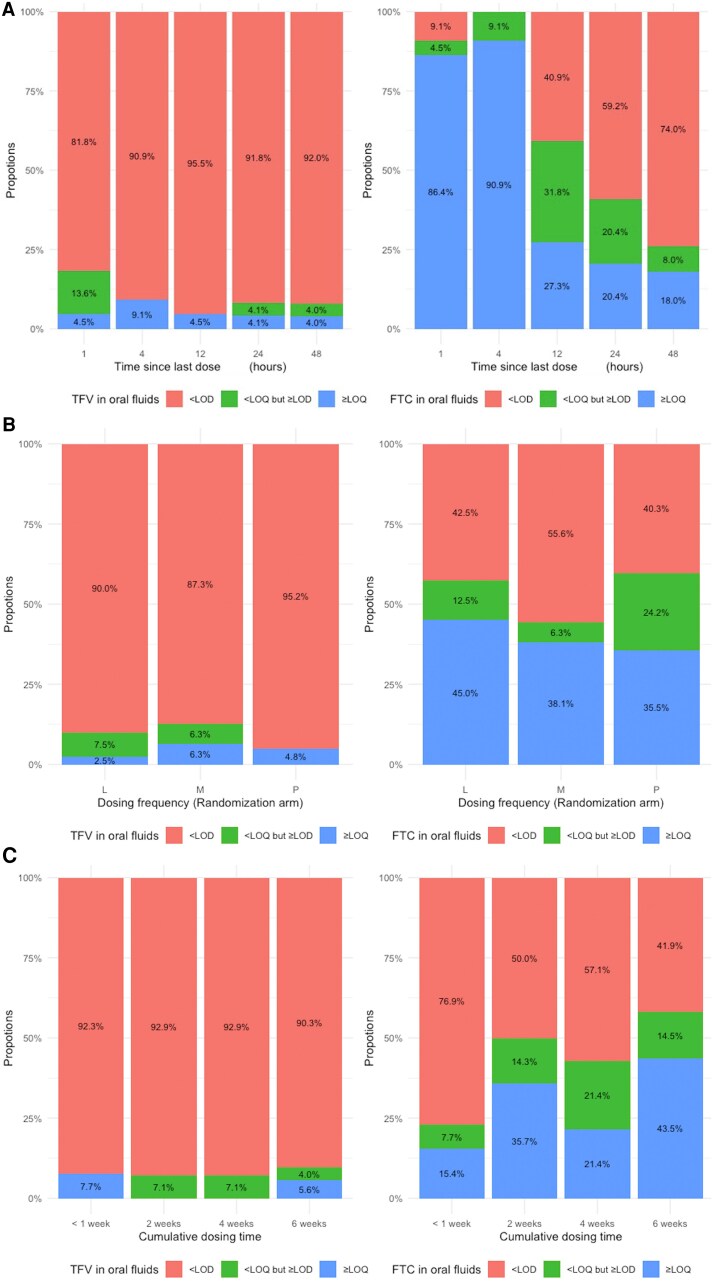
Detectability/Quantifiability of Tenofovir and Emtricitabine (TFV and FTC) in Oral Fluids by Dosing Recency (Panel A), Dosing Frequency (Panel B) and Cumulative Dosing Time (Panel C)*. (Panel A): Detectability/Quantifiability of TFV and FTC by Dosing Recency (Time Since Last Dose). (Panel B): Detectability/Quantifiability of TFV and FTC by Dosing Frequency. (Randomization arm: L-Low: 2 doses/week, M-Moderate: 4 doses/week, P-Perfect: 7 doses/week). (Panel C): Detectability/Quantifiability of TFV and FTC by Cumulative Dosing Time (weeks). *LOD, Limit of Detection (5 ng/mL); LOQ, Limit of Quantification (10 ng/mL).

Fifteen (9.0%) oral fluids in total had detectable TFV above the LOD, and eight (4.8%) were above the LOQ (Supplementary Table 3). The proportions of oral fluids with detectable TFV concentrations were 18.1%, 9.1%, 4.5%, 8.2%, and 8.0% for samples collected at 1, 4, 12, 24, and 48 hour(s) post-dose respectively (Figure 1). Detectability of TFV/FTC in oral fluids by the three dimensions of adherence was summarized in Table 1. We detected TFV only in 10% of oral fluids with a time since the last dose ≤24 hours, 5% of samples from participants randomized to the perfect adherence group (7 doses/week), and 10% of those with 6 weeks of cumulative dosing time.

**Table 1.** Detectability of Tenofovir/Emtricitabine (TFV/FTC) in Oral Fluids by Adherence (Dosing Recency vs Dosing Frequency vs Cumulative Dosing Time)

	TFV Detected in Oral Fluids (≥LOD^a^)	
***Dosing Recency***	**Yes**	**No**
Time Since Last Dose ≤ 24 h	11 (10%)	104 (90%)
24 h < Time Since Last Dose ≤ 48 h	4 (8%)	46 (92%)
***Dosing Frequency***	**Yes**	**No**
Low (2 doses/week)	4 (10%)	36 (90%)
Moderate (4 doses/week)	8 (12%)	55 (87%)
Perfect (7 doses/week)	3 (5%)	59 (95%)
***Cumulative Dosing Time (weeks)***	**Yes**	**No**
One	1 (8%)	12 (92%)
Two	1 (7%)	13 (93%)
Four	1 (7%)	13 (93%)
Six	12 (10%)	112 (90%)
***Total***	15 (9%)	150 (91%)

	FTC Detected in Oral Fluids (≥LOD^a^)	
***Dosing Recency***	**Yes**	**No**
Time Since Last Dose ≤ 24 h	75 (65%)	40 (35%)
24 h < Time Since Last Dose ≤ 48 h	13 (26%)	37 (74%)
***Dosing Frequency***	**Yes**	**No**
Low (2 doses/week)	23 (58%)	17 (42%)
Moderate (4 doses/week)	28 (44%)	35 (56%)
Perfect (7 doses/week)	37 (60%)	25 (40%)
***Cumulative Dosing Time (weeks)***	**Yes**	**No**
One	3 (23%)	10 (77%)
Two	7 (50%)	7 (50%)
Four	6 (43%)	8 (57%)
Six	72 (58%)	52 (42%)
***Total***	88 (53.3%)	77 (46.7%)

^a^LOD, Limit of Detection (5 ng/mL).

**Figure 2.**
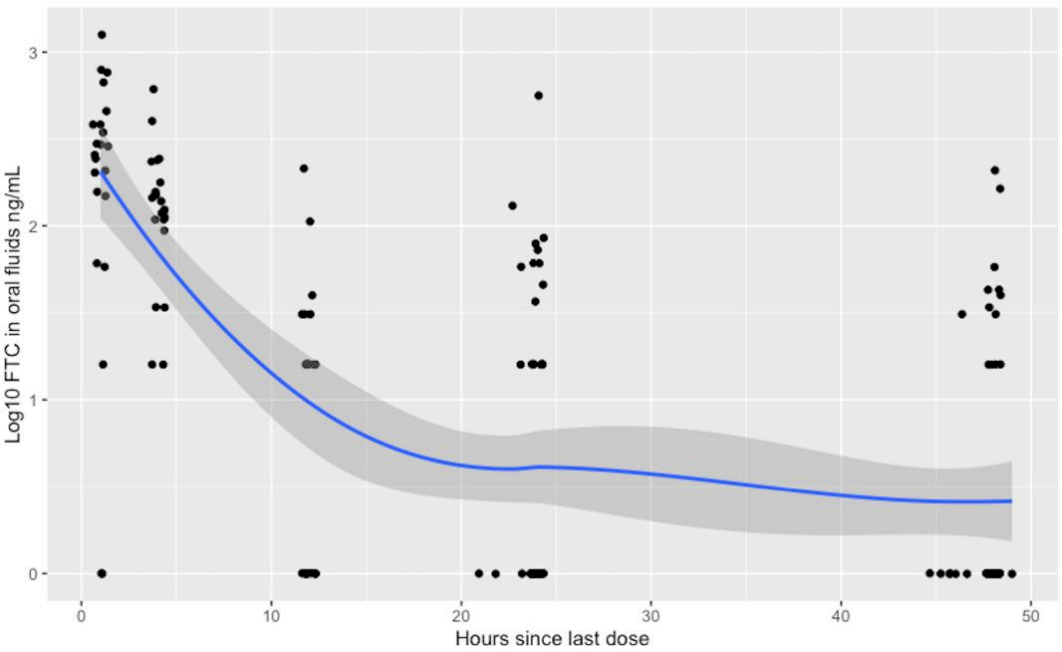
Scatterplot Emtricitabine (FTC) concentrations (Log 10 transformed) in oral fluids by dosing recency (time since last dose [hours])*. *LOESS smoothed line added with 95% confidence interval.

FTC was detectable in more oral fluid specimens and had higher drug concentrations, as compared with TFV. FTC levels in oral fluids were detected above the LOQ in 64 (38.8%) samples. Another 24 (14.5%) oral fluids had detectable FTC concentrations below the LOQ. The median (IQR) FTC concentrations were 15 (0–141) ng/mL for 115 oral fluids with time since the last dose ≤24 hours, and 0 (0–11) ng/mL for 50 oral fluids with time since last dose between 24 and 48 hours. FTC was detectable in 90.9%, 100%, 59.1%, 40.8% and 26% of oral fluids with a time since last dose at 1, 4, 12, 24, 48 hour(s) respectively (Supplementary Table 3). There was also a declining trend of mean FTC levels by dosing recency (Figure 2). Compared with samples with a time since the last dose between 24 and 48 hours, we found a higher proportion of oral fluids with a time since the last dose ≤24 hours had FTC detected (65% vs 26%).

Among 143 pairs of time-matched samples, FTC in oral fluids showed moderate correlation with FTC in plasma (0.66, 95% CI: 0.55–0.75). However, TFV in oral fluids was not correlated with TFV in plasma (0.15, 95% CI: −0.03–0.32). We also observed strong correlation between TFV and FTC in plasma (0.95, 95% CI: 0.89–0.95) but not in oral fluids (0.15, 95% CI: −0.02–0.30).

### TFV/FTC in Oral Fluids for PrEP Non-adherence

Detectability of TFV and FTC in oral fluids was not significantly associated with having more frequent doses per week or one more week of cumulative dosing time (Table 2) based on the GEE analyses. However, compared with oral fluids with a time since the last dose ≤24 hours, having a time since the last dose between 24 and 48 hours was on average associated with 79% lower odds of having detectable FTC in oral fluids (OR [odds ratio]: 0.21, 95% CI: 0.11–0.38, *P* < .01) with the same dosing frequency and cumulative dosing time.

**Table 2.** GEE Model Results for Tenofovir/Emtricitabine (TFV/FTC) Detected in Oral Fluids and Three Dimensions of PrEP Adherence^a^

GEE Coefficient	24h < Time Since Last Dose ≤48 h (vs Time Since Last Dose ≤ 24 h)	Moderate Adherence (vs Low)	Perfect Adherence (vs Low)	One More Week of Cumulative Dosing
FTC detected in oral fluids	−1.58 (−2.19 to −0.97)	0.17 (−0.88–1.21)	0.12 (−1.11–1.36)	0.15 (−0.01–0.31)
TFV detected in oral fluids	−0.64 (−2.34–0.41)	0.53 (−1.17–2.25)	−0.88 (−2.57–0.81)	0.0009 (−0.20–0.21)
GEE Odds ratio	24h < Time since last dose ≤48 h (vs Time since last dose ≤ 24 h)	Moderate Adherence (vs Low)	Perfect Adherence (vs Low)	One more week of cumulative dosing
FTC detected in oral fluids	0.21 (0.11–0.38)	1.18 (0.41–3.37)	1.13 (0.33–3.89)	1.16 (0.99–1.36)
TFV detected in oral fluids	0.53 (0.10–1.51)	1.69 (0.31–9.46)	0.41 (0.08–2.24)	1.00 (0.81–1.23)

^a^Data are presented as point estimate (95% confidence interval) unless specified otherwise.

For ROC analyses among 165 oral fluids with a time since the last dose ≤48 hours (Supplementary Table 4), the optimal FTC threshold <7.5 ng/mL has accurately classified 74% (sensitivity) samples with the last dose missed (having a time since the last dose >24 hours but ≤48 hours) as non-adherent while maintaining a moderate specificity for samples with a time since the last dose ≤24 hours (65%, 95% CI: 57%–74%). To better reflect the real-world definition of PrEP non-adherence beyond “with only one missed dose (having a time since the last dose >24 but ≤48 hours)”, we included the other 76 untested oral fluids (with 30 and 46 collected at 72 and 96 hours since the last dose, respectively). Assuming those samples had no detectable FTC, the threshold at 7.5 ng/mL achieved the same specificity (Table 3) but an improved sensitivity for recent non-adherence (90%, 95% CI [84%–94%]) and an AUC (area under the curve) of 0.79 (95% CI: 0.74–0.84).

**Table 3.** Diagnostic Accuracy of Emtricitabine (FTC) in Oral Fluids for Detecting Recent PrEP Non-Adherence (with a Time Since Last Dose >24 Hours)

FTC Thresholds in Oral Fluids (ng/mL)	Sensitivity (95% CI^a^)	Specificity (95% CI^a^)
7.5	90% (84%–94%)	65% (57%–74%)
22.5	93% (88%–97%)	48% (38%–57%)
31.5	94% (90%–98%)	45% (36%–54%)
34.4	95% (91%–98%)	44% (34%–52%)
43.5	98% (94%–100%)	42% (33%–50%)
58.5	98% (96%–100%)	39% (30%–48%)
169.5	99% (97%–100%)	20% (13%–28%)
210	100% (100%–100%)	17% (0%–24%)

^a^CI, Confidence Interval.

## DISCUSSION

In this randomized trial of directly observed FTC/TDF administration, we found that FTC detectability in oral fluids was associated with PrEP dosing recency. A threshold of FTC <7.5 ng/mL achieved high sensitivity by accurately classifying 90% of PrEP non-adherence (as early as missing one dose or with a time since the last dose >24 hours). With limited diffusion into oral fluids—possibly due to higher hydrosolubility and lower ionization [18]—TFV was present only in a few specimens and at low concentrations in oral fluids, and its detectability was not associated with dosing recency, frequency, or cumulative dosing time. Pharmacologic adherence monitoring may be valuable for detecting early signs of non-adherence and enabling timely initiation of individualized support interventions. Additionally, an objective clinical measure of non-adherence to oral pills may help some people transition to long-acting regimens, particularly when evidence of medical necessity is required for insurance coverage.

Due to improved adherence for people struggling with daily oral PrEP, long-acting injectables, such as CAB-LA or LEN, have demonstrated superiority over daily oral regimens in clinical trials [23–25]. However, it remains uncertain whether most PrEP users, who adhere well to daily oral PrEP (taking ≥4 doses/week), would still benefit from long-acting injectables for better HIV prevention [26, 27]. Therefore, the WHO recommends CAB-LA not as a replacement for daily oral PrEP but as an additional prevention option for people at substantial HIV risk [3]. Even for those facing challenges with the daily regimen, it is also unclear if CAB-LA or LEN would outperform oral daily PrEP combined with adherence monitoring and targeted interventions. Pharmacologic adherence monitoring with prompt feedback was found highly acceptable and associated with improved adherence to daily oral PrEP [10]. For example, in a cohort study among men who have sex with men (MSM), plasma TFV level feedback demonstrated modest adherence improvement [28]. Women receiving urine TFV adherence counseling also showed better adherence with higher proportions of positive urine assay results and higher levels of TFV in hair [29]. Given its wider availability, lower cost, and flexibility, daily oral of FTC/TDF, when combined with effective adherence monitoring, may achieve greater public health impact in the real-world with similar effectiveness as long-acting injectables.

With a long half-life of 17–21 days that translates to 25-fold accumulation from first dose to steady state, TFV-DP in DBS provides a significant dynamic range to differentiate varying levels of adherence over the preceding 1–3 months [14, 30]. This characteristic makes TFV-DP less susceptible to “white-coat” adherence, where consistently non-adherent users may take a dose immediately before pharmacologic testing to appear compliant [31, 32]. Therefore, TFV-DP benchmarks in DBS are often preferred for predicting cumulative adherence and explaining PrEP outcomes in trials [4, 5, 33]. However, these established benchmarks are only applicable when steady-state drug concentrations are achieved, which could be challenging in real-world settings among non-adherent users due to irregular dosing patterns [34, 35]. Also, the conservative estimation of TFV-DP benchmarks [14], using the rounded 25th percentiles of participants at each adherence level, may result in the misclassification of about 25% of participants as having lower adherence than they actually do. These limitations could partly explain the variability in observed PrEP adherence-efficacy relationships across different studies [5, 36, 37].

Unlike TFV-DP detected in DBS, FTC has a short half-life (9–10 hours) in oral fluids [20]. It does not accumulate appreciably with repeated dosing and thus indicates recent drug ingestion. While “white-coat” dosing may sometimes mask non-adherence, short half-life metrics provide valuable insights into dosing recency that does not require the attainment of steady-state. The timing of the last dose relative to high-risk behavior may be more informative than averaged dosing frequency over previous weeks for ED PrEP. A POC lateral flow assay (LFA) for urine TFV, with a cutoff of 1500 ng/mL, has shown a high specificity of 98% for accurately classifying users who took a dose within 24 hours as adherent [38]. It also has a high sensitivity of 86% for identifying non-adherence in users who missed >3 missed doses (last dose taken >96 hours prior). However, its sensitivity ranges from 16% to 52% for detecting non-adherence with 1–2 missed doses (last dose taken between 48 and 72 hours prior).

Our data demonstrated that FTC in oral fluids, using a threshold of 7.5 ng/mL, is highly sensitive for detecting PrEP non-adherence as early as one dose missed. It accurately classified 90% of users with time since last dose >24 hours as non-adherent. However, its modest specificity of 65% may lead to false positives among some adherent individuals, potentially causing unnecessary concerns. To address this limitation, a follow-up test with high specificity, such as the POC LFA for urine TFV [38], could confirm their adherent status and alleviate stress. In the development of tests for non-adherence detection (monitoring adherence), the primary goal should be to identify non-adherent users as many and as early as possible to facilitate timely interventions or regimen changes. Therefore, sensitivity should be prioritized over specificity. A highly specific but less sensitive test might misclassify non-adherent users as adherent, inadvertently reinforcing non-adherent behaviors and exposing them to increased HIV risk due to suboptimal PrEP protection.

Our study had several strengths and was one of the first randomized PK trials that comprehensively investigated the associations between TFV/FTC in oral fluids and the three dimensions of adherence. However, one limitation of our analyses is that we assumed all oral fluids with a time since the last dose >48 hours had undetectable FTC, which was supported by prior research and our pilot testing results. Considering 26% of the oral fluids with a time since last dose exactly at 48 hours had detectable FTC, it is possible that some samples, particularly those with a time since the last dose close to 48 hours post-dose, would have detectable FTC. This might lead to slightly overestimated sensitivity. Nonetheless, the effect should be minimal since all of our untested samples were collected either 72 or 96 hours since the last dose, and prior research found FTC levels to be lower than the LOQ among all oral fluids with a time since last dose ≥72 hours [20]. Reliance on the resource-intensive LC-MS/MS has also limited the real-world application potential of testing oral fluids for adherence monitoring. Further studies among real-world PrEP users with more diverse adherence patterns and oral health conditions that may affect oral fluid pharmacokinetics are needed to confirm and extend our findings.

In conclusion, we demonstrated the feasibility of detecting FTC in oral fluids to identify oral daily PrEP non-adherence. Our study found that the detectability of FTC in oral fluids is closely associated with dosing recency. A threshold of FTC <7.5 ng/mL in oral fluids demonstrated high sensitivity for identifying recent non-adherence. These findings will help establish adherence benchmarks of FTC in oral fluids and guide the development of POC tests for adherence monitoring.

## Supplementary Material

ofaf766_Supplementary_Data

## Supplementary Data

Supplementary materials are available at *Open Forum Infectious Diseases* online. Consisting of data provided by the authors to benefit the reader, the posted materials are not copyedited and are the sole responsibility of the authors, so questions or comments should be addressed to the corresponding author.
