# Supplementary material for: Oral Fluid Concentrations of Tenofovir and Emtricitabine for Monitoring HIV Antiretroviral Adherence: Findings From a Randomized Trial with Directly Observed Therapy (TARGET Study)
Source: Open Forum Infect Dis. 2025 Dec 12;13(1):ofaf766. doi: 10.1093/ofid/ofaf766 (PMC12740851; doi:10.1093/ofid/ofaf766)
Supplement: ofaf766_Supplementary_Data [file ofaf766_supplementary_data.docx]

**Supplementary Data**

**Table S1**. Participant Baseline Characteristics by PrEP Use Randomization Arms^*^

| Adherence Arm | Low  (2 doses/week) |  | Moderate  (4 doses/week) |  | Perfect  (7 doses/week) |  | All |
| --- | --- | --- | --- | --- | --- | --- | --- |
| Baseline Characteristics | (N=8) |  | (N=7) |  | (N=7) |  | (N=22) |
| Age, years | 33.5(9.3) |  | 31.1(9.4) |  | 34.4(6.6) |  | 33.1(8.1) |
| Female, N(%) | 3(38%) |  | 1(14%) |  | 5(71%) |  | 9(41%) |
| Body Mass Index, kg/m2 | 23.4(20.1-28.5) |  | 23.5(21.4-24.9) |  | 20.3(19.3-24.0) |  | 23.3(19.8-24.9) |
| Hemoglobin, g/dL | 14.7(13.7-15.0) |  | 14.4(14.4-14.9) |  | 12.3(11.2-12.7) |  | 14.2(12.5-14.7) |
| Hematocrit, % | 43.6%(41.5%-45.4%) |  | 44.1%(42.4%-45.1%) |  | 37.1%(34.6%-38.7%) |  | 42.2%(38.3%-44.7%) |
| eGFR^#^, mL/min | 109(100-116) |  | 121(104-133) |  | 91(87-97) |  | 106(91-117) |
| BUN, mg/dL | 10.6(9.4-12.7) |  | 9.7(7.7-11.3) |  | 9.3(9.1-10.2) |  | 10.0(8.8-12.0) |
| Creatinine (plasma), mg/dL | 0.91(0.82-1.03) |  | 0.82(0.78-0.91) |  | 0.73(0.68-0.88) |  | 0.83(0.74-0.96) |
| Total bilirubin, mg/dL | 0.67(0.47-0.80) |  | 0.97(0.82-1.24) |  | 0.68(0.44-0.78) |  | 0.73(0.52-0.97) |

Abbreviations: eGFR, estimated glomerular filtration rate.

^*^Data are presented as median (interquartile range or standard deviation) unless specified otherwise.

^#^Estimated with the Cockcroft-Gault equation.

**Table S2**. PrEP Adherence Patterns (Dosing Recency vs Dosing Frequency vs Cumulative Dosing time) for Oral Fluids Tested

|  | **Time Since Last Dose (Dosing Recency)** | | | | |  |
| --- | --- | --- | --- | --- | --- | --- |
| ***Adherence Arm (Dosing Frequency)*** | **1 hour** | **4 hours** | **12 hours** | **24 hours** | **48 hours** | N |
| Low (2 doses/week) | 8 | 8 | 8 | 8 | 8 | 40(24.2%) |
| Moderate (4 doses/week) | 7 | 7 | 7 | 7 | 35 | 63(38.2%) |
| Perfect (7 doses/week) | 7 | 7 | 7 | 34 | 7 | 62(37.6%) |
| N | 22 (13.3%) | 22(13.3%) | 22(13.3%) | 49(29.7%) | 50(30.3%) | 165(100%) |
|  | **Time Since Last Dose (Dosing Recency)** | | | | |  |
| ***Weeks of Dosing (Cumulative Dosing Time)*** | **1 hour** | **4 hours** | **12 hours** | **24 hours** | **48 hours** | **N** |
| <One | 0 | 0 | 0 | 6 | 7 | 13(7.9%) |
| Two | 0 | 0 | 0 | 7 | 7 | 14(8.5%) |
| Four | 0 | 0 | 0 | 7 | 7 | 14(8.5%) |
| Six | 22 | 22 | 22 | 29 | 29 | 124(75.2%) |
| N | 22 (13.3%) | 22(13.3%) | 22(13.3%) | 49(29.7%) | 50(30.3%) | 165(100%) |

**Table S3**. Tenofovir/Emtricitabine (TFV/FTC) Detected in Oral Fluids by Dosing Recency and the Limit of Detection (LOD) and the Limit of Quantification (LOQ)^*^

|  | ***TFV in oral fluids*** | | |
| --- | --- | --- | --- |
| **Time since last dose (hours)** | **<LOD** | **≥LOD but <LOQ** | **≥LOQ** |
| **1** | 18(81.8%) | 3(13.6%) | 1(4.5%) |
| **4** | 20(90.9%) | 0(0%) | 2(9.1%) |
| **12** | 21(95.5%) | 0(0%) | 1(4.5%) |
| **24** | 45(91.8%) | 2(4.1%) | 2(4.1%) |
| **48** | 46(92.0%) | 2(4.0%) | 2(4.0%) |
| All | 150(90.9%) | 7(4.2%) | 8(4.8%) |
|  | ***FTC in oral fluids*** | | |
| **Time since last dose (hours)** | **<LOD** | **≥LOD but <LOQ** | **≥LOQ** |
| **1** | 2(9.1%) | 1(4.5%) | 19(86.4%) |
| **4** | 0(0%) | 2(9.1%) | 20(90.9%) |
| **12** | 9(4.1%) | 7(31.8%) | 6(27.3%) |
| **24** | 29(59.2%) | 10(20.4%) | 10(20.4%) |
| **48** | 37(74.0%) | 4(8.0%) | 9(18.0%) |
| All | 77(46.7%) | 24(14.5%) | 64(38.8%) |

^*^LOD=5 ng/mL; LOQ=10 ng/mL

**Table S4**. Diagnostic Accuracy of Emtricitabine (FTC) in Oral Fluids for Differentiating Samples “with a time since Last dose ≤24 hours” from Samples “with a time since last dose <48 but > 24 hours”

| FTC threshold in oral fluids (ng/mL) | Sensitivity (95%CI) | Specificity(95%CI) |
| --- | --- | --- |
| 7.5 | 74%(62%-86%) | 65%(57%-74%) |
| 22.5 | 82%(70%-92%) | 48%(38%-57%) |
| 31.5 | 86%(76%-94%) | 45%(36%-54%) |
| 34.4 | 88%(78%-96%) | 44%(34%-52%) |
| 43.5 | 94%(86%-100%) | 42%(33%-50%) |
| 58.5 | 96%(90%-100%) | 39%(30%-48%) |
| 169.5 | 98%(94%-100%) | 20%(13%-28%) |
| 210 | 100%(100%-100%) | 17%(0%-24%) |
